# T-cell receptor activation-associated cytokine release is impaired in septic patients with faecal peritonitis

**DOI:** 10.1186/cc13415

**Published:** 2014-03-17

**Authors:** DG Gore, L Preciado-Llanes, GH Mills, AW Heath, RC Read

**Affiliations:** 1 Sheffield Teaching Hospitals NHS Foundation Trust, Sheffield, UK; 2 The University ofSheffield, UK; 3 University of Southampton, UK

## Introduction

Sepsis is associated with immune hyporesponsiveness but the immunological processes behind this are ill defined.

## Methods

This study quantified differences in plasma concentrations of cytokines between septic patients with faecal peritonitis, age and gender-matched surgical patients (without sepsis) and age and gender-matched healthy participants. In addition, cytokine levels were measured in supernatant from peripheralblood mononuclear cells stimulated with anti-CD3 and anti-CD3+anti-CD28, incubated for 4 days. Cytokine concentrations of IL-1β, IL-5, IL-6, IL-8, IL-10, IL-13, IL-17A, IFNγ and TNFα were determined by multiplex cytometric bead array.

## Results

**Figure 1:**
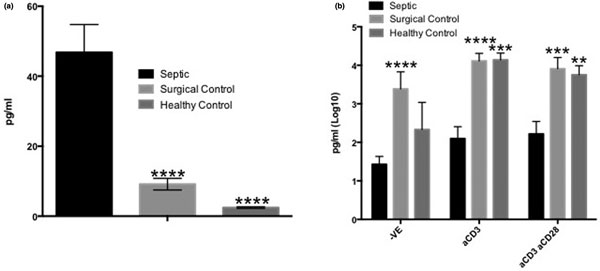
**Concentration of IL-6 in plasma on day 0 (a) and supernatant on day 4 (b)**.

Plasma levels of IFNγ and IL-13 were lower in septic patients compared with healthy participants. In contrast, plasma levels of IL-6 (see Figure 1) and IL-8 were increased in septic patients compared with both surgical patients and healthy participants. Plasma levels of IL-10 were significantly higher only in comparison with surgical patients. Following incubation with anti-CD3 and anti-CD3+anti-CD28, concentrations of IL-1β, IL-5, IL-6 (see Figure 1), IL-13, IL-17A, IFNγ and TNFα were markedly decreased in samples from septic patients. In addition, stimulation with anti-CD3+anti-CD28 resulted in lower production of IL-10 in septic patients. Lower concentrations of IL-8 were detected in septic patient samples stimulated with only anti-CD3. We found cytokine levels of IL-12p70 remained unaffected across all groups and stimuli.

## Conclusion

We demonstrated a proinflammatory cytokine profile in blood from septic patients, preceding a pan downregulation of all assessed cytokines following in vitro T-cell stimulation. To our knowledge, this study is the first to perform an immune functional assay across these three groups.

